# Textbook outcome in gastrectomy: useful metric or moving target? A scoping review

**DOI:** 10.1007/s10120-025-01659-x

**Published:** 2025-09-26

**Authors:** Riadh Salem, Lorenzo Giorgi, Wing K. Chou, Sheraz R. Markar

**Affiliations:** 1 Surgical Intervention Trials Unit, Nuffield Department of Surgical Sciences, University of Oxford, Oxford, UK; 2 Department of Surgery, Churchill Hospital, Oxford University Hospitals NHS Trust, Oxford, UK

**Keywords:** Textbook outcome, Gastrectomy, Surgical quality

## Abstract

**Background:**

Composite metrics including Textbook Outcome (TO) and Textbook Oncological Outcome (TOO) are increasingly utilised to assess quality in gastric cancer surgical research. However, inconsistent and variable reporting limits their clinical application.

**Objective:**

This scoping review aimed to catalogue definitions and criteria of TO and TOO in gastrectomy, report achievement rates and determinants, associations with survival outcomes, and identify methodological gaps.

**Methods:**

A search was conducted in MEDLINE, Embase, Web of Science, and Scopus from inception to April 2025. Eligible studies reported TO or TOO for adults undergoing curative-intent gastrectomy for cancer. Reviewers screened studies and extracted data on characteristics, definitions, achievement rates, and survival outcomes. Owing to heterogeneity, findings were summarised narratively.

**Results:**

Forty-five studies (published 2017–2025; *n* = 139,972 patients) were included. Definitions varied, with 26 unique components identified. Common components were adequate lymphadenectomy (≥ 15 nodes), absence of postoperative complications (Clavien–Dindo grade ≥ II), and no 30-day readmission. Median TO and TOO achievement rates were 58.6% (IQR: 37.6–75.8) and 30.3% (IQR: 23.6–40.2). The primary barriers were inadequate lymphadenectomy and CD ≥ II complications. Twelve studies reported a significant association between TO/TOO and improved overall and disease-free survival. Influencing factors included age, comorbidity, tumour characteristics, surgeon volume, and surgical approach. Limitations included non-standardised definitions, limited patient-reported outcomes, and a lack of prospective validation.

**Conclusion:**

TO and TOO are associated with improved survival in gastrectomy but are hampered by inconsistent definitions and limited prospective evidence. Standardisation, patient-reported outcomes, and prospective validation are needed to realise their potential as clinically useful quality metrics.

**Supplementary Information:**

The online version contains supplementary material available at 10.1007/s10120-025-01659-x.

## Introduction

Robust quality metrics are essential in surgical oncology to accurately capture the multifaceted nature of patient care [1]. Traditional measures, like mortality or morbidity rates, often fail to reflect the entirety of a patient’s clinical journey and its oncological success [2]. Recently, composite outcome measures have gained popularity. Textbook Outcome (TO) and its oncological version, Textbook Oncological Outcome (TOO), have emerged as significant benchmarks in the evaluation of gastric cancer surgery, to really provide a more global composite perspective of outcomes from gastric cancer surgery.

TO was first proposed by Dijs-Elsinga et al. as a patient-preferred quality-of-care metric, defined by an uncomplicated postoperative course without readmissions, re-operations, adverse events, and with a hospital stay no longer than average [3]. The concept was later formalised by Kolfschoten et al. in 2013 within colorectal surgery, where it was proposed as a composite measure representing the “ideal” surgical episode [4].

The value of TO as a quality measure was recognised and adopted for other oncological surgeries, including those of the oesophagus and stomach. A key development came with the Dutch Upper Gastrointestinal Cancer Audit (DUCA), which adapted TO for oesophagogastric surgery, recognising the need for procedure-specific definitions [5]. The DUCA group defined TO using a set of ten criteria: (1) complete (potentially curative) resection; (2) negative microscopic margins; (3) evaluation of ≥ 15 lymph nodes; (4) absence of intra-operative complications; (5) no severe postoperative complications (Clavien–Dindo grade ≥ II); (6) no re-intervention; (7) no unplanned intensive care admission; (8) length of stay ≤ 21 days; (9) no 30-day readmission; and (10) no 30-day mortality. Building on this, TOO emerged [6, 7], incorporating not only peri-operative measures but also the administration and completion of guideline-concordant systemic therapy. Importantly neither textbook outcome includes any measure of patient health related quality of life (HRQL) despite this being widely recognised as a hugely important outcome of cancer surgical treatment for patients.

Despite growing interest in TO and TOO for gastrectomy, definitions remain inconsistent and outcomes variable. The aim of this scoping review is to: (1) catalogue published definitions and criteria of TO and TOO in gastrectomy; (2) map their reported incidence and determinant factors; (3) synthesise evidence linking TO and TOO achievement with survival outcomes; and (4) identify methodological and thematic gaps to guide future research.

## Methods

The methodology for this scoping review was developed in accordance with the Preferred Reporting Items for Systematic Reviews and Meta-Analyses extension for Scoping Reviews (PRISMA-ScR) [8]. A scoping review was chosen to map the breadth of definitions, determinants, and knowledge gaps related to TO and TOO. This approach is ideal for surveying the landscape of existing research rather than quantifying specific outcome effects. Preliminary searches confirmed significant heterogeneity in TO/TOO criteria and reported outcomes, precluding a quantitative synthesis. This methodology was therefore selected to collate the current evidence, identify opportunities for consensus, and prioritise future research.

Studies were eligible if they reported either TO or TOO for adult human patients (aged ≥ 18 years), with confirmed gastric cancer. Included patients must have undergone a curative intent gastrectomy (total or subtotal) using any surgical approach, including open, laparoscopic, or robotic techniques. Only original research articles published in English and in peer-reviewed journals were included.

A comprehensive literature search was conducted on 10th April 2025 across four databases from inception to 5 April 2025: MEDLINE (PubMed), Embase (Ovid), Web of Science, and Scopus. For completeness, the reference lists of all included studies and relevant reviews were manually screened for additional eligible articles.

The search strategy was developed iteratively and tailored to each database using controlled vocabulary (e.g. MeSH terms) and relevant keywords, including terms such as “gastrectomy,” “textbook outcome,” “surgical quality,” and “survival.” The complete search string used for PubMed database is available in the supplement.

All search results were imported into Bookends reference management software, where duplicate records were automatically identified and removed. Titles and abstracts were independently screened by two reviewers (RS, LG) using predefined inclusion criteria. Full-text articles were then retrieved and assessed for eligibility. Disagreements at any stage were resolved through discussion, with unresolved cases adjudicated by a third reviewer (SRM).

A structured data extraction form was developed in Microsoft Excel to ensure consistency across studies. It captured key study characteristics, patient demographics, cancer stage distribution, surgical approach and extent of resection, definitions and criteria of TO/TOO, rates of TO/TOO achievement, and survival outcomes stratified by TO/TOO status. Data were extracted independently by two reviewers (RS, LG), and a random 10% of the extracted data underwent a quality audit by a third reviewer (WKC) to ensure accuracy and consistency.

Primary outcomes were the frequency of individual TO/TOO components and study-specific definitions. Secondary outcomes included the rates of TO and/or TOO achievement and their association with overall survival (OS) and disease-free survival (DFS). The final version of the extraction form is available upon request.

A formal risk of bias was not conducted, in keeping with the objective of mapping the existing evidence rather than evaluating the strength of findings. Limitations of studies, including issues of generalisability, confounding, and definition heterogeneity, are addressed narratively in the Discussion section.

Due to anticipated heterogeneity in the definitions of TO and TOO, as well as variation in the reporting of survival outcomes, a meta-analysis was not pursued. Findings are presented in narrative format and supported by summary tables.

## Results

### Study selection

**Fig. 1:**
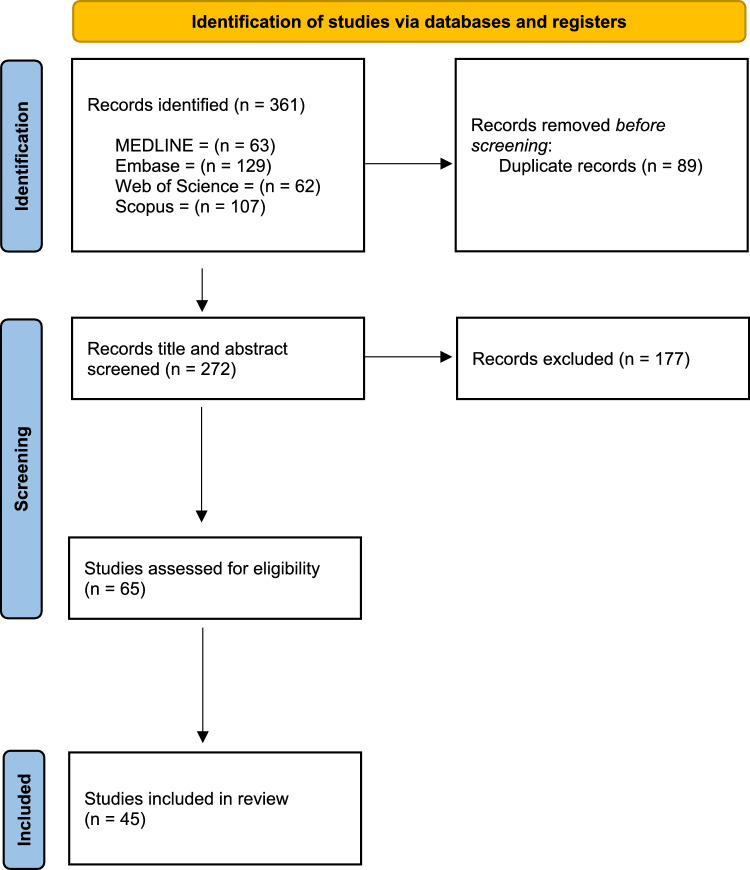
The PRISMA flow diagram outlining the study selection process

The systematic search yielded 361 records; after de-duplication, 242 unique citations remained and were screened by title and abstract. Of these, 177 were excluded, leaving 65 articles for full-text review. 45 studies met all eligibility criteria and were included in the final synthesis. The study selection process is summarised in the PRISMA flow diagram (Fig. 1). Reasons for exclusion at the full-text stage included: lack of TO/TOO reporting, or non-original, non-peer reviewed research.

### Study characteristics

The 45 included studies [5, 7, 9–51] were published between 2017 and 2025 and covered a broad geographic distribution, including Europe (35%), East Asia (33%), America (24%), and Australasia (8%). Most studies were retrospective cohort or registry-based designs, with only four prospective studies and no randomised controlled trials. The majority (73%) included both total and subtotal gastrectomy. Surgical approaches were reported in 75% of studies, with open surgery reported in 20 studies, 26 reported laparoscopic gastrectomy, and 9 included robotic approaches. Several studies reported more than one surgical approach. Most studies enrolled patients with undifferentiated cancer clinical stages, although the reporting of stage distribution was heterogeneous. Study-level characteristics are summarised in Table 1.

**Table 1:** Characteristics of studies reporting textbook outcome and textbook oncological outcome after gastric cancer surgery

Study	Country	Design	Patients N	Gastrectomy	Surgical approach*		TO achieved (%)	Too achieved (%)
Busweiler 2017 [5]	Netherlands	Cohort retrospective	1772	TG, SG	Open	74%	569 (32.1)	–
					MIS	25%		
van der Kaaij 2018 [51]	Netherlands	Cohort retrospective	105	TG, SG	Open	100%	48 (45.7)	–
van der Werf 2019 [50]	Netherlands	Cohort retrospective	2943	TG, SG	Open	65%	1029 (35)	–
					MIS	35%		
Priego 2019 [49]	Spain	Cohort prospective	96	TG, SG	Open	51%	49 (51)	–
					Lap	49%		
Levy 2019 [48]	Canada	Cohort retrospective	1836	Not reported	NR	402 (21.9)	–	
Levy 2020 [47]	Canada	Cohort retrospective	1660	Not reported	NR	378 (22.8)	–	
Narendra 2021 [46]	Australia	Cohort retrospective	796	Not reported	NR	300 (37.6)	–	
Aquina 2021 [45]	USA	Cohort retrospective	22,085	TG, SG	NR	–	7034 (31.8)	
Voeten 2021 [44]	Netherlands	Cohort retrospective	3690	TG, SG	Open	NR	1412 (38.2)	-
					Lap	NR		
Tian 2021 [43]	Australia	Cohort retrospective	1253	TG, SG	NR	426 (33.9)	–	
Bolger 2021 [42]	Ireland	Cohort retrospective	258	TG, SG	Open	51%	97 (37)	–
					Lap	49%		
Roh 2021 [41]	Korea	Cohort prospective	395	TG	Robotic	50%	284(71.9)	–
					Lap	50%		
Cibulas 2022 [40]	USA	Cohort retrospective	34,688	TG, SG	NR	–	8249 (23.8)	
Spolverato 2022 [39]	Italy, USA	Cohort retrospective	910	TG, SG	NR	–	302 (33.2)	
Dal Cero 2022 [38]	Spain	Cohort retrospective	1293	TG, SG	Open	64.2%	531 (41.1)	–
					Lap	35.8%		
Levy 2022 [37]	Canada	Cohort retrospective	1836	Not reported	NR	402 (21.9)	–	
Chen 2022 [36]	China	Cohort retrospective	3993	TG, SG	Open	12.4%	3361 (84.2)	–
					Lap	87.6%		
Sędłak 2022 [35]	Poland	Cohort retrospective	194	TG, SG	NR	–	78 (40.2)	
Hirata 2023 [34]	USA	Cohort retrospective	161	TG, SG	Open	75%	NR^§^	
					Lap	25%		
Sędłak 2023 [7]	Europe	Cohort retrospective	1700	TG, SG	Open	NR	1164 (68.5)	388 (22.8)
					MIS	NR		
Çetinkaya-Hosgör 2023 [33]	Germany	Cohort retrospective	99	TG	Open	15.4%	52 (52.5)	
					Lap	84.6%		
Morito 2023 [32]	Japan	Cohort retrospective	141	TG, SG	Open	38%	73 (52%)	–
					Lap	62%		
Carbonell Morote 2023 [31]	Spain	Cohort retrospective	91	TG, SG	NR		31 (34.1)	–
D’Souza 2023 [30]	New Zealand	Cohort retrospective	64	TG, SG	Open	88%	17 (26.5)	–
					Lap	12%		
Bouffler 2024 [29]	Australia	Cohort retrospective	136	TG, SG	Open	82.3%	84 (62)	–
					Lap	17.6%		
Lin 2024 [28]	China	Cohort retrospective	2658	TG, SG	Open	19.3%	1770 (66.6)	–
					Lap	57.9%		
					Robotic	22.8%		
Zhong Zi 2024 [27]	China	Cohort retrospective	1389	TG, SG	Open	NR	1112(80.1)	–
					Lap	NR		
Xue 2024 [26]	China	Cohort retrospective	527	TG, SG	Robotic	100%	430 (81.6)	–
Lin 2024 [25]	China	Cohort retrospective	1540	TG, SG	Lap	100%	994 (64.5%)	–
Avila 2024 [24]	USA	Cohort retrospective	21,015	TG, SG	Open	72.8%	–	5903 (28)
					Lap	21.9%		
					Robotic	5.3%		
Rawicz-Pruszyński 2024 [23]	USA	Cohort retrospective	13,885	TG, SG	Open	66.2%	–	4209 (34.7)
					Lap	22.2%		
					Robotic	11.6%		
Tu 2024 [22]	China	Cohort retrospective	3674	TG, SG	Lap	100%	2883 (78.4)	-
Velayudham 2024 [21]	UK	Cohort retrospective	312	TG, SG	Open	53.3%	160 (51.2)	–
					Lap	26.9%		
Flemming 2024 [20]	Germany	Cohort retrospective	44	TG, SG	Lap	68.1%	24 (54.4)	–
					Robotic	31.8%		
Lin G 2024 [19]	China	Cohort retrospective	291	TG, SG	Lap	66.6	234 (80.4)	–
					Robotic	33.3		
Wei 2024 [18]	China	Cohort retrospective	218	TG, SG	Lap	50%	172 (78.8)	–
					Robotic	50%		
De Jongh 2024 [17]	Europe	Cohort prospective	759	TG, SG	Robotic	100%	525 (69)	–
Huang 2024 [16]	China	Cohort retrospective	3626	TG, SG	Open	NR	2737 (75.4)	2089 (57.6)
					Lap	NR		
Realis Luc 2024 [15]	Italy	Cohort retrospective	300	TG, SG	Open	NR	176 (58.7)	71 (33.3)
					Lap	NR		
Ramos 2024 [14]	Brazil	Cohort retrospective	681	TG, SG	Open	61.7	444 (65.2)	–
					MIS	21.8		
Oh 2024 [13]	Korea	Cohort retrospective	4902	SG	Lap	96.9	3736 (82.9)	–
					Robotic	3.1		
Sun 2024 [12]	China	Cohort retrospective	585	TG, SG	Lap	100%	NR^§^	
Zhong 2024 [11]	China	Cohort prospective	206	CG	Open	77.6%	162 (78.7)	–
					Lap	22.5%		
Pelc 2025 [10]	Europe, US	Cohort retrospective	193	TG, SG	NR	-	29 (15)	
Zhong 2025 [9]	China	Cohort retrospective	972	TG, SG	NR	-	653 (67.1)	

^*^Proportion of total cases performed using each approach, when reported

^§^Overall TO achievement not reported by the authors

Abbreviations: *TO* textbook outcome, *TOO* textbook oncological outcome, *TG* total gastrectomy, *SG* subtotal gastrectomy, *CG* completion gastrectomy, *MIS* minimally invasive surgery, *Open* open approach, *Lap* laparoscopic approach, *Robotic* robotic-assisted approach, % percentage of the study cohort achieving the stated endpoint, *NR* not reported

### Definitions and variability

There was a substantial variation in how TO and TOO were defined across the included studies. In total, 26 unique components were identified across all definitions. The most commonly used composite was that proposed by Busweiler et al. [5]; this has appeared in 9 studies. No single component was reported by all studies (Table S3). The most frequent component was the evaluation of ≥ 15 lymph nodes, which appeared in 93% of studies. Other commonly used components included no readmission within 30 days of surgery (88.8%) and a hospital stay of ≤ 21 days (88%). A detailed breakdown of component definitions and their frequency is provided in Table 2.

**Table 2:** Reporting frequency of textbook outcome and textbook oncological outcome criteria in gastric cancer surgery

TO component	N of studies	% of 45
Curative resection	20	44.4%
No intra‑operative complication^§^	26	57.7%
*Tumour‑negative margins*		
↳ Local pathology report	18	40.0%
↳ ACP definition^+^	19	42.2%
↳ RCP definition^+^	5	11.1%
*Lymph nodes resected*		
↳ ≥ 15	42	93.3%
↳ ≥ 16	2	4.4%
↳ ≥ 25	1	2.2%
*No post-operative complications*		
↳ Clavien–Dindo ≥ II	27	60.0%
↳ Clavien–Dindo ≥ III	12	26.6%
*No re‑intervention*		
↳ ≤ 30 d	36	80.0%
↳ ≤ 90 d	2	5.0%
No ICU readmission	31	68.8%
No ICU readmission ≤ 30 d	1	2.0%
No prolonged ICU stay (> 48 h)	6	13.3%
*No prolonged stay*		
↳ ≤ 14 d	1	2.2%
↳ ≤ 19 d	1	2.2%
↳ ≤ 21 d	37	88.0%
↳ 75th percentile	4	9.3%
*No post‑op mortality*		
↳ ≤ 30 d	35	77.7%
↳ ≤ 90 d	6	13.3%
*No hospital readmission*		
↳ ≤ 30 d	40	88.8%
↳ ≤ 90 d	2	4.4%
Minimally invasive approach	44	2.2%

TOO component	N of studies	% of 11
*Appropriate peri‑operative systemic therapy*		
↳ Neo + Adjuvant	9	81.8%
↳ Adjuvant only	1	9%

Columns show the number of studies (*N*) that included the component and the corresponding percentage of all TO-eligible studies (denominator = 45) or TOO-eligible studies (denominator = 11)

Sub-components are indented beneath their parent criterion

^§^Defined as any deviation from the ideal intraoperative course, such as intraoperative transfusion, unintended adjacent organ injury or resection, and conversion from minimally invasive to open surgery for any reason

+ ACP classifies R1 when tumour is present at the resection margin, whilst the RCP defines R1 as Tumour within 1 mm of the margin

Abbreviations: *TO* textbook outcome, *TOO* textbook oncological outcome, *ACP* Association of Clinical Pathologists margin definition, *RCP* Royal College of Pathologists margin definition, *CD* Clavien–Dindo postoperative complication grade, *ICU* intensive care unit, *d* days, *h* hours

Definitions of TOO showed similar variability. Most studies included both pre- and postoperative guideline-concordant treatment as part of the definition. One study reported only adjuvant chemotherapy [9], whilst another did not include any systemic oncological therapy at all despite calling it TOO [10].

### Achievement of textbook outcome

A total of 139,972 patients were analysed across the 45 included studies. Amongst these, 46,030 patients from 37 studies were assessed for TO, of whom 26,842 achieved TO. This represents an overall pooled achievement rate of 56%. The median TO rate was 58.6% (IQR: 37.6–75.8). In contrast, eleven studies assessed TOO, encompassing 99,568 patients, with 29,005 (29.13%) achieving TOO. The median TOO achievement rate was 30.3% (IQR: 23.6–40.2).

### Components limiting TO and TOO achievement

Despite heterogeneity in definitions, consistent patterns were observed in terms of which individual component most commonly limited TO and TOO achievement. Inadequate lymphadenectomy consistently emerged as the most frequent limiting factor, with studies reporting 30% compliance in some cohorts [42]. Absence of major postoperative complications was another critical determinant, with CD ≥ II being the most common barrier to TO in several studies [37, 40, 42]. In TOO-specific analyses, failure to receive guideline-concordant chemotherapy (whether neoadjuvant, adjuvant, or both) was amongst the least commonly fulfilled criteria and significantly limited TOO attainment [16]. Additionally, prolonged length of hospital stay and unplanned readmission, though less frequently highlighted, were also frequently unmet criteria, particularly when stringent thresholds (e.g. ≤ 14 or ≤ 7 days) were applied [28, 32].

### Association of textbook outcome with survival

12 studies have demonstrated a significant association between achieving TO or TOO and improved overall and disease-free survival. Whilst follow-up periods varied, the survival benefit was consistently observed across 1-, 3-, and 5-year outcomes. Patients who met TO or TOO criteria had notably higher survival rates and lower hazard ratios compared to those who did not. A detailed summary of these findings is presented in Table 3.

**Table 3:** Survival impact of achieving a textbook (oncological) outcome

	Follow-up	Outcome	TO/TOO achieved (%)	TO/TOO not achieved (%)	HR (95% CI)	*p*-value
van der Kaaij 2018 [51]	3 years	OS	74	45	2.58 (1.25–5.32)*	0.018
van der Werf 2019 [50]	3 years	OS	64	42	0.62 (0.54–0.71)	< 0.001
Levy 2019 [48]	3 years	OS	75	55	0.59 (0.48–0.72)	< 0.001
Dal Cero 2022 [38]	3 years	OS	73	53	0.67 (0.55–0.83)	< 0.001
Levy 2022 [37]	3 years	OS	75	55	0.59 (0.48–0.72)	< 0.001
Çetinkaya 2023 [33]	2 years	OS	98	84	0.13 (0.02–0.68)	0.02
	2 years	DFS	69	54	0.44 (0.21–0.95)	0.04
Lin 2024 [28]	5 years	OS	65	40	0.49 (0.43–0.55)	< 0.001
	5 years	DFS	63	38	0.49 (0.43–0.54)	< 0.001
Zhong zi 2024 [11]	5 years	OS	62	49	1.47 (1.24–1.73)*	< 0.001
Lin 2024 [25]	5 years	OS	64	42	0.51 (0.44–0.60)	< 0.001
	5 years	DFS	62	40	0.51 (0.44–0.60)	< 0.001
Velayudham 2024 [21]	3 years	OS	61	47	0.63 (0.43–0.91)	0.014
	3 years	DFS	59	45	0.64 (0.44–0.91)	0.013
Huang 2024 [16]	5 years	OS	61	45	0.67 (0.61–0.74)	< 0.001
	5 years	DFS	57	44	0.73 (0.66–0.81)	< 0.001
Pelc 2025 [10]	5 years	OS	50	30	NR	0.002

^*^For studies that originally reported the risk associated with not achieving TO, HRs were inverted (1/HR) so that all values consistently reflect the effect of achieving TO. This adjustment applies to van der Kaaij 2018 and Zhong 2024, which originally presented HRs for failure to achieve the outcome

Abbreviations**:** *TO* textbook outcome, *TOO* textbook oncological outcome, *OS* overall survival, *DFS* disease-free survival, *HR* hazard ratio, *CI* confidence interval, *NR* not reported

Studies analysing individual TO and TOO components demonstrate that negative resection margins (R0 resection) consistently show a survival advantage, with hazard ratios (HR) ranging from 0.62 to 0.63 (p < 0.001) [7, 28, 37, 38, 40]. Adequate lymphadenectomy (≥ 15 lymph nodes) also showed a significant association with reduced mortality in several studies [28, 37, 40]. Cibulas et al. showed a significant association with reduced risk of mortality (HR 0.79; *p* < 0.001) [40]. However, this association was not consistently observed across all studies, with some reporting no significant relationship [7, 35, 51]. The absence of severe postoperative complications (CD ≥ II) improves survival [7, 28, 30, 35, 38, 50, 51]: for example, patients without CD ≥ II complications had substantially better overall survival (HR 0.49, 95% CI 0.29–0.81, *p* = 0.006) [51]. Similarly, avoiding prolonged hospital stay (≤ 21 days) and unplanned ICU admission were independently associated with improved outcomes [37].

### Patient, hospital, and treatment factors associated with textbook outcome

A range of patient-related factors have been shown to influence the likelihood of achieving TO or TOO. Reported negative predictors include older age (particularly ≥ 75 years) [7, 11, 15, 16, 28, 33, 34, 38–40, 42, 45], higher comorbidity burden (e.g. elevated Charlson Comorbidity Index or ASA status) [7, 9, 15, 38, 49], and adverse tumour characteristics such as larger size, higher stage [38], or proximal tumour location [28, 37]. Additional factors associated with reduced TO or TOO attainment include significant preoperative weight loss [9] and low haemoglobin [38].

Hospital-related factors play a significant role in the likelihood of achieving TO or TOO. Higher volume and surgical experience were frequently associated with improved TO rates. Studies showed that high-volume centres more often achieve composite outcomes and specific quality metrics, such as adequate lymph node retrieval [5, 43, 46, 50]. However, this association is not universally observed; for instance, the PRESTO study in Ontario did not find a significant link between hospital or surgeon volume and overall TO achievement. However, they did find that hospital volume was associated with adequate lymphadenectomy [47].

Several treatment-related factors significantly influence the likelihood of achieving TO or TOO. Minimally invasive surgery, such as laparoscopic and robotic surgery, is consistently associated with higher TO/TOO attainment, likely due to lower complication rates, adequate lymph node dissection, and shorter hospital stays [24, 42]. Some authors have considered adding MIS as a separate component of TO [27]. In contrast, open surgery and conversion from minimally invasive to open are independently linked to TO failure [33]. Whilst most sources support a positive association between MIS and TO/TOO, one study noted that laparoscopic gastrectomy favoured non-TO in their unit, potentially due to technical complexity, low case numbers, and a learning curve [21]. Subtotal gastrectomy is generally more likely to result in TO than total gastrectomy, which carries higher technical demands and complication rates [15, 28, 36]. Intraoperative factors such as prolonged operative time and increased blood loss are also associated with TO failure [25, 32]. Finally, Enhanced Recovery After Surgery (ERAS) protocols, when adhered to, improve TO rates [33], whilst compliance is influenced by surgical approach and extent of resection [15].

## Discussion

This scoping review synthesises evidence from 45 primary studies published between 2017 and 2025 investigating the definitions, determinants, and achievement rates of Textbook Outcome for gastric cancer surgery. Despite considerable international interest, our review highlights profound heterogeneity in how TO and TOO are defined and measured, with no universally accepted criteria. Data demonstrate that only a modest proportion of patients achieve TO (median 59%) or TOO (median 30%). The achievement of TO/TOO is consistently associated with improved long-term survival, reinforcing their relevance as quality indicators for clinicians, hospital leaders, and policymakers. Still, they remain subject to definitional inconsistency, methodological challenges, and a limited integration of patient-centred outcomes, all of which constrain the use of TO/TOO as routine benchmarks in gastric cancer surgery.

A systematic scoping review by Gregersen et al. examined TO and TOO in oesophagogastric cancer surgery [59]. Whilst covering both oesophageal and gastric procedures, their findings for gastric cancer offer a useful comparison and external validation for our results. They also found significant heterogeneity in TO and TOO definitions, supporting our key conclusion on the lack of standardisation. Importantly, their reported median TO (56.5%) and TOO (31.9%) rates align closely with ours (58.6% and 30.3%), reinforcing the reliability of these figures and highlighting the need for a universal definitional framework.

### Strengths of textbook outcome

A major advantage of TO and TOO is their ability to provide a multidimensional assessment of surgical quality. Unlike traditional single-metric endpoints such as mortality or individual complications, these composite measures encapsulate the entire perioperative journey: from technical success (e.g. no intraoperative complication, R0 resection, adequate lymphadenectomy) to smooth postoperative recovery. This is especially relevant to gastrectomy, an advanced high-risk procedure with multiple potential pitfalls, as it allows for a broader measure of what constitutes ‘ideal’ care [5]. By combining technical, pathological, and recovery criteria, TO/TOO offer a comprehensive view of surgical performance that is more reflective of day-to-day clinical complexity.

Perhaps the most compelling advantage of TO/TOO is their robust and reproducible association with improved long-term survival. Across diverse settings, patients achieving TO or TOO consistently demonstrate better overall and disease-free survival, even after accounting for confounding variables such as age, comorbidity burden, and tumour stage [10, 16, 21, 28, 33, 37, 38, 48, 50, 51]. This prognostic value elevates TO/TOO from retrospective quality audit tools to clinically meaningful indicators. This will help clinicians in assessing treatment efficacy and patients understanding potential long-term benefits.

TO/TOO provides a valuable benchmarking tool, enabling comparisons of surgical quality across different surgeons or institutions, as well as different surgical techniques or perioperative protocols [52]. When consistently defined, these metrics can identify high-performing centres and enable the dissemination of best practices. Their composite nature provides a more equitable basis for comparison than isolated outcomes, supporting targeted quality improvement initiatives. Moreover, the intuitive notion of a ‘textbook outcome’ resonates with patients and the public, potentially enhancing transparency and trust in surgical services [53]. For researchers, TO/TOO offers robust composite endpoints for evaluating new techniques or technologies, encapsulating the multidimensional facets of surgical interventions [54, 55]. For example, a new surgical technique might not significantly alter mortality in isolation but could lead to a higher TO rate by reducing complications or shortening hospital stay.

### Limitations and challenges

Despite their strengths, the current use of TO and TOO in gastrectomy is restricted by some considerable shortcomings. The most critical drawback is the absence of uniformity in its definition. There is no consensus on which components should be included, how they are measured, or what cut-offs constitute a ‘success’. This definitional variability impedes meaningful comparisons across studies, institutions, and regions, and precludes robust meta-analysis or the establishment of universal benchmarks. In the absence of a common framework, the scientific and practical utility of TO/TOO remains limited.

The ‘all-or-nothing’ approach further complicates interpretation. By requiring that all predefined criteria be met to classify an outcome as ‘textbook’, this method may mask clinically relevant gradations of success and penalise minor, non-critical deviations. This is particularly problematic as it equates rare but catastrophic events (e.g., mortality) with more common and arguably less severe deviations, such as a modest delay in discharge. This issue is compounded by the lack of consensus on component definitions, particularly for postoperative complications. The choice of threshold (whether Clavien-Dindo grade ≥ II or ≥ III constitutes a failure) fundamentally alters the metric’s clinical relevance and prognostic power. Emerging evidence suggests a definition using a CD ≥ III cut-off may be more strongly associated with long-term survival [60], implying that the inclusion of minor complications can dilute the prognostic signal of the composite outcome. For a complex procedure like gastrectomy, this strict dichotomy can lead to low achievement rates that may be misinterpreted as poor quality care, when in fact the majority of patients experience good, albeit not “perfect,” results. This oversimplification may thus mask important clinical details and inadequately represent the spectrum of patient experiences [56].

Another important challenge is the inadequate adjustment for patient heterogeneity. TO/TOO achievement is strongly influenced by baseline factors such as age, comorbidities, nutritional status, and tumour characteristics [31, 41]. Crude, unadjusted comparisons risk conflating institutional or surgeon performance with differences in case-mix, particularly disadvantaging centres caring for higher-risk populations. The lack of validated risk-adjustment models for TO/TOO in gastrectomy limits true benchmarking and quality assessment.

A further conceptual limitation and major critique is the discordance between clinician-derived TO/TOO definitions and patient-reported outcomes (PROs). Existing frameworks are largely built around technical or pathological perspective, often overlooking domains of functional recovery and quality of life that matter most to patients. For TO/TOO to become a truly patient-centred quality metric, the integration of PROs is essential. This gap highlights that current TO/TOO frameworks may not fully reflect the"success"of surgery from the patient’s lived experience.

Another limitation is the lack of prospective validation, particularly within the context of randomised controlled trials (RCTs). To date, most of the evidence supporting the adoption of TO and TOO in gastric cancer surgery is derived from retrospective or observational studies. The integration and assessment of TO/TOO as endpoints in RCTs would not only yield higher levels of evidence regarding their prognostic value, but would also facilitate the evaluation of interventions aimed at improving these composite outcomes. Such studies are critical to establishing the clinical utility and generalisability of TO/TOO as robust quality metrics in gastrectomy.

Finally, the generally low and variable achievement rates pose interpretational challenges. While reflecting the aspirational nature of a ‘perfect’ outcome, low rates may reduce discriminatory power and practical relevance, particularly if the reasons for non-achievement are diverse or minor [57]. This lack of uniformity stems from the differing definitions, patient populations, and types of healthcare settings. Moreover, significant international variations, with some East Asian centres reporting higher TO rates than many Western counterparts, raise questions about the global applicability of a single TO standard [58]. If meeting an “ideal” outcome consistently remains the reality for a small portion, its utility as a universal benchmark requires careful consideration and may require stratification or adjustment.

### Research gaps and future directions

Several important gaps need to be addressed to enhance the validity and impact of TO/TOO in gastrectomy. Arguably, the most crucial is the urgent need for a nationally/internationally endorsed, standardised definition that balances technical, oncological, and recovery criteria. It also needs to be adaptable to diverse healthcare contexts. Achieving consensus through methodologies such as Delphi processes, with multidisciplinary and patient input, is a critical next step. Without standardisation, it is impossible to enable meaningful benchmarking, research synthesis, or quality improvement.

Equally important is the integration of patient-reported outcomes and quality of life domains into TO/TOO frameworks. Future research should prioritise the identification, validation, and routine collection of PROs relevant to post-gastrectomy recovery, ensuring that composite metrics genuinely reflect what patients’ value most in their care. Based on established evidence of the post-gastrectomy patient experience and international consensus, future frameworks should incorporate, at a minimum, domains from the GASTROS core outcome set for gastric cancer surgery trials [61]. Validated instruments, such as the EORTC QLQ-STO22 and the FACT-Ga, are available to measure these domains and could serve as the foundation for a truly patient-centred TO. The inclusion of these domains is not merely an addition of variables but a fundamental step toward redefining surgical success to align with the patient’s lived experience [62].

Methodological innovations are also required to address the limitations of the current binary framework. Future research should move beyond the ‘all-or-nothing’ approach by developing and validating more nuanced scoring systems. These could include weighted TO scores, where components are assigned importance based on their impact on long-term survival or patient-reported quality of life, or graded/ordinal TO scores, which would create a hierarchy of outcomes from ideal to most severe, allowing for a more granular assessment of quality and targeted improvement efforts. Furthermore, the determination of optimal cut-off values for individual components must be grounded in robust evidence. Consensus-driven methodologies, such as a Delphi process involving both clinicians and patients, should be used to establish clinically meaningful definitions for components like ‘prolonged stay’ or ‘severe complication’. For continuous variables, such as the number of lymph nodes retrieved, statistical methods should be employed to identify data-driven thresholds that best predict long-term oncological outcomes.

Addressing regional disparities in TO/TOO achievement and understanding the underlying drivers are important for global quality improvement. Comparative studies employing standardised definitions, have the potential to identify best practices, as well as inform the development of region-specific standards.

Finally, future research should explore the association between TO/TOO and broader long-term outcomes beyond survival, including functional recovery, nutritional status, chronic symptom burden, and overall quality of life. Implementation science studies are needed to assess how TO/TOO can be effectively integrated into clinical practice, public reporting, and policy, as well as their potential utility in surgical training, accreditation, and health system evaluation.

## Financial support

This research was supported by the Royal College of Surgeons of England pump priming grant and the National Institute for Health Research.

## Supplementary Information

Below is the link to the electronic supplementary material.

Supplementary file1 (DOCX 582 KB)

## Data Availability

Data sharing requests will be considered by the writing group upon written request to the corresponding authors.
